# Mild Maternal Obstructive Sleep Apnea in Non-obese Pregnant Women and Accelerated Fetal Growth

**DOI:** 10.1038/s41598-018-29052-y

**Published:** 2018-07-17

**Authors:** Ayana Telerant, Galit Levi Dunietz, Ariel Many, Riva Tauman

**Affiliations:** 1 Sleep Disorders Center, Tel Aviv Medical Center, Tel Aviv University, Tel Aviv, Israel; 2 Department of Obstetrics and Gynceology, Lis Maternity Hospital, Tel Aviv Medical Center, Tel Aviv, Israel; 3 Sackler School of Medicine, Tel Aviv University, Tel Aviv, Israel; 4 Sleep Disorders Center, Department of Neurology, University of Michigan, Ann Arbor, Michigan USA

## Abstract

The prevalence of obstructive sleep apnea (OSA) during pregnancy is rising. OSA during pregnancy has been associated with hypertensive disorders of pregnancy and gestational diabetes. The effect of maternal OSA on the fetus, particularly on fetal growth, is less apparent. Most of the currently available human data is based on non-objective assessment of OSA and includes heterogeneous populations with inadequate control on confounders, such as maternal obesity and pregnancy complications. Using objective tools in non-obese women with uncomplicated pregnancies, we aimed to investigate the association between maternal OSA and fetal growth. A total of 155 non-obese pregnant women were recruited. Birth-weight percentile of the newborns of women with mild OSA was significantly higher compared with the newborns of non-OSA controls (72% vs. 57%, respectively, *P* < 0.01). Birth-length and triceps thickness measurements were significantly higher among the newborns of women with OSA compared with controls (*P* = 0.02 for both). The proportion of large for gestational age (LGA) newborns was higher among women with OSA compared with controls (28% vs. 8%, respectively, *P* = 0.04). Our results suggest that maternal OSA during the third trimester of pregnancy - even in a mild form -is associated with accelerated fetal growth.

## Introduction

Obstructive sleep apnea (OSA) is characterized by repetitive episodes of upper airway obstruction during sleep, resulting in disruption of ventilation, hypoxemia, and sleep fragmentation. OSA has a significant impact on public health, and may lead to daytime sleepiness, reduced neurocognitive functions, accidents, and metabolic and cardiovascular consequences if left untreated^1^. Various intermediary mechanisms link OSA to these morbidities including sympathetic activation, endothelial dysfunction, oxidative stress, inflammation and metabolic dysregulation^2^. Over the past several decades, the prevalence of OSA has been continuously rising in the general population, most likely due to the growing obesity epidemic^1^. Accumulating evidence shows that habitual snoring, the hallmark symptom of OSA, increases in frequency during pregnancy and affects up to one-third of women by the third trimester^3–9^. Similar to the general population, overweight and obesity increase the risk of OSA in pregnant women^10–12^, and the prevalence of maternal OSA is rising, with an annual increase of 24%^13,14^. OSA during pregnancy has been associated with adverse maternal outcomes, including hypertensive disorders of pregnancy^9,13–21^ and gestational diabetes^15–22^.

The effect of maternal OSA on the fetus, particularly on fetal growth, is less apparent. Animal studies results are inconsistent, with some demonstrating that maternal intermittent hypoxia, a model of OSA, leads to low birthweight of the pups^23–25^. A recent publication has shown that late gestational intermittent hypoxia induces metabolic dysfunction, as reflected by increased body weight and adiposity index in male mice offspring when they reach adulthood^26^, suggesting long-term metabolic dysregulation in offspring exposed to intermittent hypoxia in pregnancy. Indeed, several downstream effects of OSA may affect placental function and fetal growth and not always in the same direction.

Numerous human studies have shown an association between maternal OSA and adverse fetal outcomes, such as intrauterine growth restriction, low Apgar scores, preterm births and neonatal intensive care unit admissions^17,27–29^. Studies on the association between maternal OSA and birthweight, however, yielded conflicting results^3,27,30–37^. Taken together, most of the currently available human data were reported in studies that used non-objective tools for the assessment of OSA and included heterogenous populations with inadequate control on confounders, such as maternal obesity and adverse maternal pregnancy complications that could potentially affect fetal growth. We conducted a prospective cohort study for assessing the association between maternal OSA and fetal growth with good control on maternal obesity and pregnancy complications.

## Methods

Women in the third trimester of a singleton, uncomplicated pregnancy who attended a low-risk obstetric surveillance outpatient clinic at the Noy Women Health Care Center or the outpatient clinics at the Lis Maternity Hospital were recruited between June 2008 and December 2010. At enrollment (gestational week 25–27), all participants completed a questionnaire on smoking exposure history, medical and obstetric history and current pregnancy complications. They also responded to questions on the presence of habitual snoring before and during pregnancy, including the Berlin Questionnaire^38^ and the Epworth sleepiness scale (ESS)^39^. All participants underwent an ambulatory overnight sleep study between 33 to 36 weeks of gestation in which the watch-PAT 200 device (Itamar Medical; Israel) was used. The device had been validated in pregnancy and shown to correlate well with polysomnography^40^.

Women with an apnea hypopnea index (AHI) >5 per hour of sleep were considered to have OSA. We used the standard adult criteria for OSA, since there is no definitive threshold for OSA in pregnancy, and decided to restrict our analysis to the mild form of OSA. A medical records review was conducted by one of the researchers who was unaware of the sleep study results. Pertinent demographic (gender, gestational age, birthweight), and clinical information (mode of delivery, Apgar scores at 1 and 5 minutes, and any perinatal complications) was collected. The neonatal birthweight percentile was calculated according to gender and gestational age using a birth centile curves reference standard from Israel^41^. Small for gestational age (SGA) was defined as a birth percentile <10^th^ percentile, and large for gestational age (LGA) was defined as a birth percentile >90^th^ percentile for age and gender.

Body length, head circumference and adiposity (skinfold thickness measurements of the subscapular and triceps areas) were measured at birth in a subset of newborns. Recumbent crown-heel length was measured on a length board (O’Leary Premie Length Board, Ellard Instrumentation Ltd., Monroe, WA). Fronto-occipital head circumference was measured using a standard 1-cm wide measuring tape. Skinfold thickness measurements were performed using Holtain calipers (Holtain, UK). Each measurement was recorded twice and the average was used for analysis^42,43^.

The study was approved by the institutional review board of the Tel Aviv Medical Center. All methods were performed in accordance with the relevant guidelines and regulations. Informed consent was obtained from all participants and/or their legal guardians.

### Statistical analysis

Descriptive statistics, chi-square tests, logistic and linear regression analysis were used to compare the distributions of maternal and newborn characteristics in non-obese women with mild OSA (AHI within a range of 5–14/h) compared with controls (AHI < 5). We examined the association between mild maternal OSA exposure (using an AHI < 5 as the referent group) and newborn size (first defined as birthweight percentile [continuous measure) and then as a binary variable) by classifying newborns with a birthweight percentile >90 as LGA vs. appropriate for gestational age newborns (birthweight percentile ≤90). We examined these associations by constructing bivariate and adjusted linear and logistic regression models controlled for parity and pre-pregnancy body mass index (BMI). Since common pregnancy complications that are known predictors of newborn birthweight percentiles, i.e., gestational diabetes and gestational hypertensive disorders of pregnancy, were absent in our sample, they were not included as covariates in our regression models. SAS 9.4 (Cary, NC) was used for these analyses.

## Results

**Figure 1:**
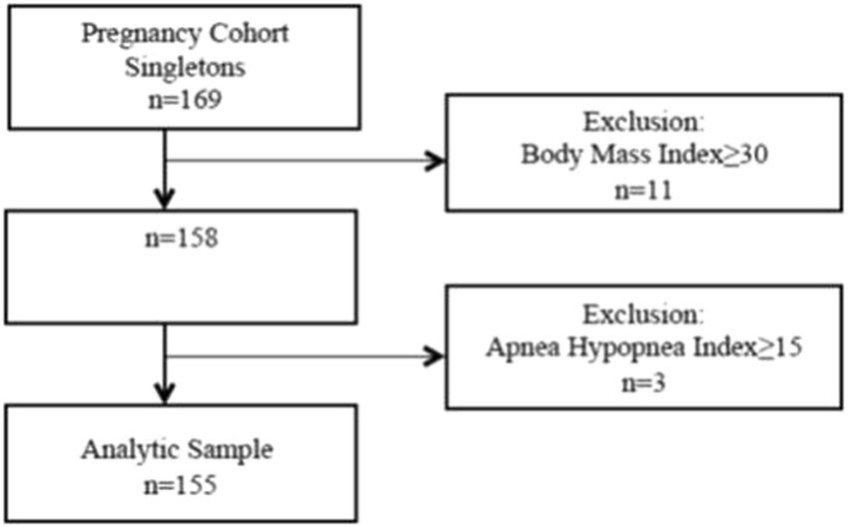
Recruitment Flowchart.

A total of 169 women were recruited, of whom 11 had a pre-pregnancy BMI >30 kg/m^2^ and were excluded from the study. Of the remaining 158, only three women had moderate-to-severe OSA (an AHI >15/h) and therefore were also excluded. The final analytic sample included 155 non-obese pregnant women with mild OSA (Fig. 1) of whom 26 (17%) had mild OSA. None of the participants developed hypertension, preeclampsia or gestational diabetes mellitus throughout the entire pregnancy. There were no pre-term deliveries in the study group, and one delivery at a gestational age of 36 weeks in the control group.

The maternal and pregnancy characteristics of the women with mild OSA and the controls are presented in Table 1. They were similar for the two groups with the exception of the mean pre-pregnancy BMI, which was significantly higher in the women with OSA compared with the controls, although the mean weight gain rate (kg/week) was similar. As expected, significantly more women with OSA had a positive Berlin score. No differences in ESS scores were found between the two groups.

**Table 1:** Maternal and Sleep Study Characteristics of 155 Non-obese, Non-hypertensive, and Non-diabetic Pregnant Women with Mild Obstructive Sleep Apnea.

Variable	Mild OSA (n = 26)	Controls (n = 129)	*P*
Mean maternal age, years (SD)	34 (4.3)	33 (4.1)	0.2
Nulliparous (%)	13 (54)	60 (47)	0.6
Mean pre-pregnancy BMI (SD)	24 (2.9)	22 (2.7)	0.02
Mean weight gain rate (kg/week)	0.4 (0.2)	0.4 (0.1)	0.9
Smokers (%)	3 (13)	4 (3)	0.1
Mean Epworth sleepiness scale (SD)	9.3 (5.3)	8.5 (4.2)	0.4
Positive Berlin score (%)	7 (27)	15 (12)	0.04
Mean apnea hypopnea index	8.7 (3)	1.3 (1.4)	<0.01
Mean SpO2	95.1 (1.3)	96.0 (1.0)	<0.01
SpO2 nadir	88.6 (20.2)	92.4 (1.4)	0.4

OSA, obstructive sleep apnea; SD, standard deviation; BMI, body mass index.

**Figure 2:**
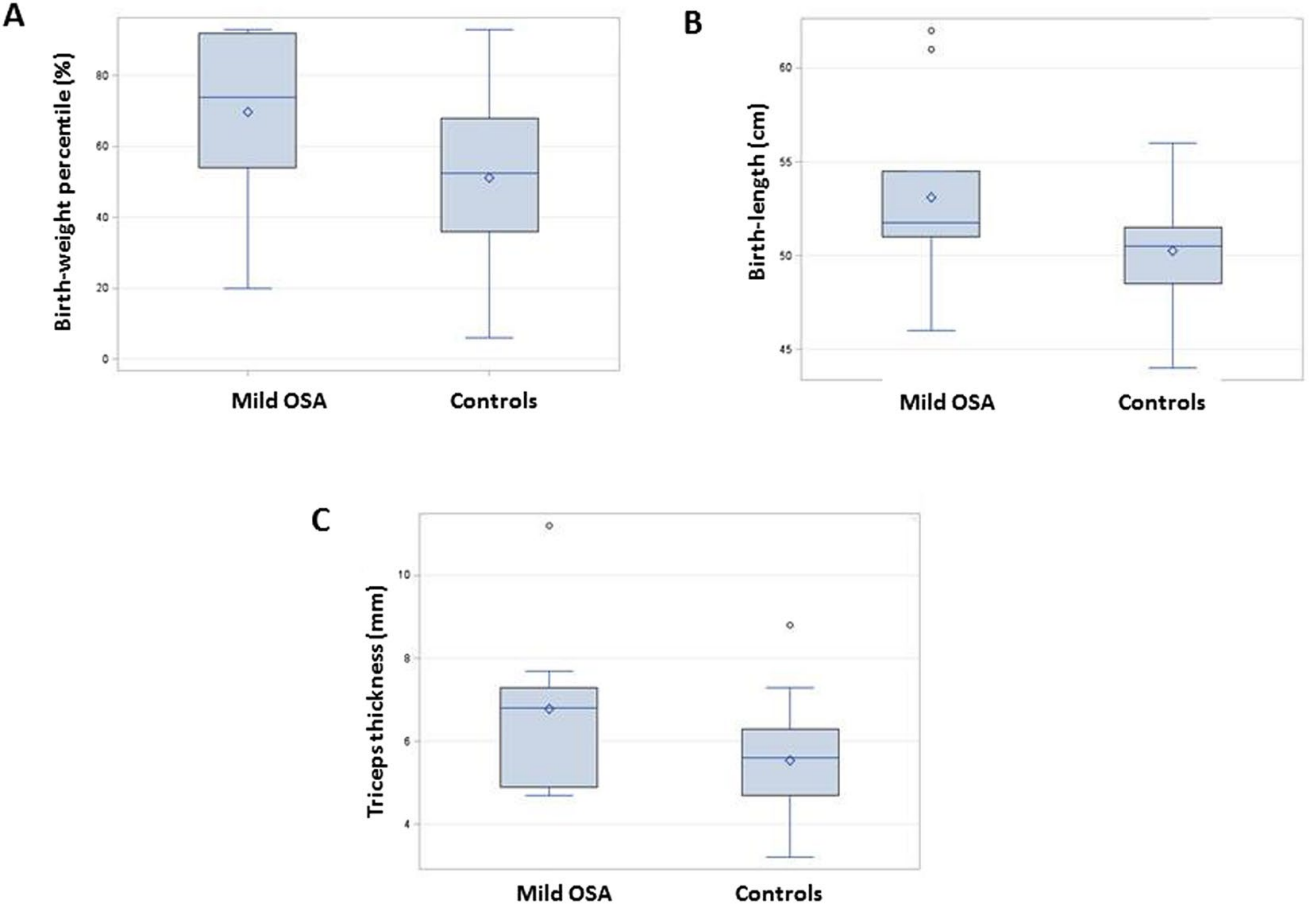
(**A**) Birth-weight percentile of newborns of mothers with mild OSA (n = 26) compared to newborns of controls (n = 129), (p < 0.01). (**B**) Birth-length of newborns of mothers with mild OSA (n = 26) compared to newborns of controls (n = 129), (p = 0.02). (**C**) Triceps thickness of newborns of mothers with mild OSA (n = 26) compared to newborns of controls (n = 129), (p = 0.02).

The delivery and newborn characteristics are presented in Table 2. The mean birthweight percentile of newborns of women with mild OSA was significantly higher compared with the newborns of the controls (72% vs. 57%, respectively, *P* < 0.01, Fig. 2A). The proportion of LGA newborns was significantly higher among the women with OSA compared with the controls (28% vs. 8%, respectively, *P* = 0.04). The percentage of SGA among women with OSA did not differ from that of the controls. Moreover, in a subset of 80 newborns, the mean birth length and mean triceps thickness measurements were significantly higher among the newborns of women with OSA compared with controls (*P* = 0.02 and *P* = 0.02, Fig. 2B,C, respectively). The mean 1-minute Apgar score was significantly lower among newborns of women with mild OSA (*P* < 0.01), and the proportion of Apgar score <7 at 1 minute was significantly higher (23% vs. 5%, respectively, *P* < 0.01).

**Table 2:** Delivery and Newborn Characteristics in a Cohort of Non-Obese, Non-Hypertensive, and Non-Diabetic Women with Mild Obstructive Sleep Apnea.

Variable	Mild OSA (n = 26)	Controls (n = 129)	*P*
Mean gestational age at birth, weeks (SD)	39.3 (1.2)	39.3 (1.2)	0.8
Mean birthweight percentile (SD)	71 (23)	57 (24)	<0.01
Small for gestational age (%)	2 (5)	1 (2)	0.3
Large for gestational age (%)	7 (28)	10 (8)	0.04
Mean head circumference, cm (SD)^*^	34.6 (1.5)	34.6 (1.3)	0.99
Mean birth length, cm (SD)^*^	53.1 (4.6)	50.3 (2.6)	0.02
Mean subscapular thickness, mm^*^	5.4 (1.2)	5.1 (1.1)	0.5
Mean triceps thickness, mm^*^	6.8 (2.0)	5.5 (1.3)	0.02
Male newborn	15 (58)	71 (55)	0.6
Mode of delivery (%)^*^			
Spontaneous vaginal	18 (74)	95 (76)	0.4
Cesarean section	6 (26)	23 (18)	
Vacuum	0 (0)	8 (6)	
Mean 1-minute Apgar score (SD)	8.3 (1.3)	8.9 (0.7)	<0.01
1-minute Apgar score ≤7 (%)	6 (23)	7 (5)	<0.01
Mean 5-minute Apgar score (SD)	9.7 (0.6)	9.8 (0.4)	0.07
5-minute Apgar score ≤7 (%)	0	0	—
Mean cord blood PH (SD)	7.3 (0.1)	7.3 (0.2)	0.3

OSA, obstructive sleep apnea; SD, standard deviation. ^*^Calculated for a subgroup of 80 women.

Bivariate and multivariate linear regression analyses were conducted with the birthweight percentile as the dependent variable, and the results are shown in Table 3. After adjustment for parity and pre-pregnancy BMI, the birthweight percentile of newborns of the women with mild OSA was 16.5 units higher compared with newborns of the controls. Bivariate and multivariate logistic regression analyses were conducted with LGA as the dependent variable and the findings are shown in Table 4. After adjustment for parity and pre-pregnancy BMI, the women with mild OSA had increased odds for an LGA newborn compared with the controls (OR = 5.1, 95% CI 1.3, 20.0).

**Table 3:** Association between Maternal Apnea-Hypopnea Index and Newborn Birthweight Percentiles in A Cohort of Non-Obese Women with Mild Obstructive Sleep Apnea.

Parameter	Model 1: Bivariate			Model 2: Multivariate		
	Regression Coefficient	SE	*P*	Regression Coefficient	SE	*P*
AHI ≥ 5	14.4	5.1		16.5	5.4	
AHI < 5	[Ref]	[Ref]	0.01	[Ref]	[Ref]	<0.01
Nulliparous				[Ref]	[Ref]	
Multiparous	—	—		8.9	4.0	0.03
Pre-pregnancy Body mass index	—	—		1.9	0.7	<0.01

AHI, apnea hypopnea index; SE, standard error; Model 1, bivariate linear regression with birthweight percentile as the dependent variable; Model 2. multivariate linear regression with birthweight percentile as the dependent variable and AHI as the independent variable, adjusted for parity and pre-pregnancy body mass index.

**Table 4:** Association between Apnea-Hypopnea Index and Large for Gestational Age Babies in a Cohort of Non-Obese Women with Mild Obstructive Sleep Apnea OSA

	Large for Gestational Age			
	Model 1: Bivariate		Model 2: Multivariate	
	Odds Ratio	95% CI	Odds Ratio	95% CI
AHI ≥ 5	4.5	1.5, 13.3	5.1	1.3, 20.1
AHI < 5	[Ref]	[Ref]	[Ref]	[Ref]
Nulliparous	—	—	[Ref]	[Ref]
Multiparous			3.7	0.9, 15.9
Pre-pregnancy Body mass index	—	—	1.2	1.0, 1.5

AHI, apnea hypopnea index; CI, confidence interval; Model 1, bivariate multinomial logistic regression with categorical newborn size large for gestational age as the dependent variable; Model 2, multivariate multinomial logistic regression with categorical newborn size large for gestational age as the dependent variable and AHI as the independent variable, adjusted for parity and pre-pregnancy body mass index.

## Discussion

The mild form is the most frequent presentation of OSA in non-obese otherwise healthy pregnant women. An association between mild maternal OSA in these women and fetal growth has not been investigated before. The present study is the first to demonstrate that mild maternal OSA in non-obese pregnant women with no gestational diabetes or hypertensive disorders of pregnancy is associated with accelerated fetal growth that is expressed in several dimensions of growth such as weight, length and adiposity and that it increases the risk of LGA newborns. This cohort limited to non-obese women with no pregnancy complications enabled us to examine the associations of the mild form of OSA on fetal growth and avoid potential confounders, such as maternal obesity and adverse maternal pregnancy complications that could potentially affect fetal growth.

OSA in the general population is associated with a variety of metabolic disturbances, including glucose intolerance, insulin resistance and lipid dysregulation^44^. While it is well established that normal pregnancy is associated with physiological alterations in glucose and lipid metabolism^45–47^, and that maternal OSA may be associated with an increased risk of gestational diabetes mellitus^15–22^, little information is available on the metabolic consequences of OSA during pregnancy and their effect on fetal growth. Indeed, several downstream effects of OSA, such as intermittent hypoxia, increased sympathetic activity, repeated arousals, repeated episodes of elevated blood pressure and peripheral vasoconstriction, potentiation of inflammatory cascades and metabolic alteration, may affect placental function and fetal growth in directions yet to be established.

The effect of maternal OSA on fetal growth has been studied, but the results were conflicting. The results of the present study support previous publications that have also shown association between maternal OSA and LGA infants^35–37^. However, most of the currently available human data were based on studies that used non-objective tools for the assessment of OSA and included heterogenous populations with inadequate control of the above-cited confounders. Those factors alone may explain the conflicting results. It is also possible that different components of OSA (intermittent hypoxia/sleep fragmentation) or the severity of OSA (mild vs. moderate-severe) affect placental function and fetal growth in different ways.

Our study results are in contrast to those recently published by Pamidi *et al*. who also included a relatively healthy non-obese cohort of pregnant women^34^. Their study showed that maternal OSA was associated with an increased risk of delivering an SGA baby. Similar to our cohort, most of their OSA cases in the third trimester were mild and characterized by sleep fragmentation rather than by oxygen desaturations, as has been demonstrated previously in pregnancy^5,8,48,49^. However, the main disparity between their study and ours that could explain the different results is in the different study designs. Pamidi *et al*. focused solely on OSA and SGA and recruited only women with a third-trimester ultrasound that showed <75^th^ centile predicted weight in order to minimize the number of LGA babies in their cohort, thus excluding the possibility of examining a link between OSA and accelerated fetal growth. By not being restricted by any fetal growth criteria, our study design could better reflect the effect of maternal OSA on fetal growth. Moreover, our findings on increased birth length and adiposity in a subset of newborns supported our observation of accelerated fetal growth in women with mild OSA.

Since parity and maternal BMI are known risk factors for accelerated fetal growth and LGA, we controlled for both variables and found that mild maternal OSA is an independent predictor of increased birthweight and LGA.

Several mechanisms may explain our results, among them maternal lipids levels, fetal insulin and placental function. It is possible that the metabolic consequences associated with even mild OSA contribute to or exaggerate the normal metabolic alterations present in late gestation, thus creating a metabolic milieu favorable for accelerated fetal growth.

All our participants were non-obese women, however, the ones who had a higher BMI (still within the normal range) tended to develop OSA during pregnancy, although the rate of their weight gain did not differ from that of the controls. Also, similar to the general population, smoking tended to be more prevalent among women with OSA, although not to a level of significance. In support of a previous publication^50^, our results also indicated that the Berlin questionnaire and the Epworth sleepiness scale perform poorly in the setting of pregnancy.

Finally, the mean 1-minute Apgar score was significantly lower among newborns of women with OSA compared with controls and, similar to previous publications, the proportion of low Apgar scores (Apgar score <7) was higher among women with OSA^3,16^.

The strengths of our study include its prospective nature and relatively large number of participants in the third trimester of pregnancy who underwent objective sleep studies within a narrow window of pregnancy (weeks 33–36). Further strengths include a homogeneous population of non-obese pregnant women with no pregnancy complications and the addition of an analysis of other measures of fetal growth, such as head circumference, birth length and adiposity. The major limitation of this study is the usage of an ambulatory sleep study rather than polysomnography, which enables better detection of flow limitation and sleep fragmentation. Another limitation is the lack of information regarding paternal anthropometry which was found to be associated with fetal growth^51,52^.

In summary, birthweight is a marker of fetal outcome and a surrogate indicator of conditions occurring in the intrauterine environment. It has been suggested that the impact of birthweight echoes throughout the course of life and may influence the risk of cardiovascular disease, diabetes, and obesity (“fetal programming”). Our results suggest that maternal OSA in pregnancy — even in a mild form — is associated with accelerated fetal growth that is expressed in several dimensions of growth such as weight, length and adiposity. These findings have significant clinical implications since accelerated fetal growth may affect the health of both the mother and the baby and may predispose the baby to abnormal growth later in life. Studies exploring the mechanisms underlying this association are warranted. Since OSA is a treatable condition, interventional trials involving the treatment of maternal OSA are also recommended.
